# Insights into *Bactericera cockerelli* and *Candidatus* Liberibacter solanacearum interaction: a tissue-specific transcriptomic approach

**DOI:** 10.3389/fpls.2024.1393994

**Published:** 2024-08-30

**Authors:** Mohan Singh Rajkumar, Freddy Ibanez-Carrasco, Carlos A. Avila, Kranthi K. Mandadi

**Affiliations:** ^1^ Texas A&M AgriLife Research and Extension Center, Weslaco, TX, United States; ^2^ Department of Entomology, Texas A&M University, College Station, TX, United States; ^3^ Department of Horticultural Sciences, Texas A&M University, College Station, TX, United States; ^4^ Department of Plant Pathology & Microbiology, Texas A&M University, College Station, TX, United States; ^5^ Institute for Advancing Health Through Agriculture, Texas A&M AgriLife, College Station, TX, United States

**Keywords:** *Bactericera cockerelli*, *Candidatus* Liberibacter solanacearum (*C*Lso), RNA-Seq, coexpression, ovaries, salivary glands

## Abstract

The tomato-potato psyllid, *Bactericera cockerelli* (Šulc), belonging to the Hemiptera order, is an insect pest of solanaceous crops and vectors a fastidious bacterium, *Candidatus* Liberibacter solanacearum (*C*Lso), the presumptive causal agent of zebra chip and vein greening diseases in potatoes and tomatoes, respectively. The genome of *B. cockerelli* has been sequenced recently, providing new avenues to elucidate mechanistic insights into pathogenesis in vegetable crops. In this study, we performed RNA-sequencing of the critical psyllid organs (salivary glands and ovaries) involved in *C*Lso pathology and transmission to host plants. Transcriptome analysis revealed differentially expressed genes and organ-specific enrichment of gene ontology (GO) terms related to metabolic processes, response to stress/stimulus, phagocytosis, proteolysis, endocytosis, and provided candidate genes encoding transcription factors (TFs). To examine gene regulatory networks across the psyllid organs under *C*Lso(-) and *C*Lso(+) conditions, we performed weighted gene co-expression network analysis (WGCNA), and unique modules differentiating the psyllid organs were identified. A comparative GO analysis of the unique gene modules revealed functional terms enriched in response to stress, gene regulation, and cell division processes in the ovaries. In contrast, respiration, transport, and neuronal transmission-related GO terms were enriched in the salivary glands. Altogether, this study reveals new insights into tissue-specific expression of the psyllid organs in the absence or presence of *C*Lso bacterium. This knowledge can be leveraged to develop new pest and disease management strategies by delineating the regulatory networks involved in the psyllid-*C*Lso interaction.

## Introduction

The tomato-potato psyllid, *Bactericera cockerelli* (Hemiptera: Triozidae), is an important insect pest among several vegetable crops, including tomatoes, potatoes, peppers, and eggplants (Wenninger and Rashed, 2024). The growing prevalence and spread of *B. cockerelli* in several American and Oceanian countries, especially in the face of global climate changes, is concerning (Wan et al., 2020; Mora et al., 2021). *B. cockerelli* transmits *Candidatus* Liberibacter solanacearum (*C*Lso), the presumptive causal agent of potato zebra chip and tomato vein greening diseases (Mora et al., 2021). This insect feeds on the phloem tissues of plants to obtain nutrients and essential amino acids. Not only the host plants are adversely damaged by feeding-associated injury and nutrient depletion (Valenzuela et al., 2020; Mora et al., 2021), the transmission of *C*Lso bacterium during feeding further aggravates plant health by inducing various disease symptoms, including chlorosis, necrosis, upward leaf curing, and stunting (Munyaneza, 2012; Avila et al., 2019; Mora et al., 2021). The interactions and relationship between the psyllid and bacterium have yet to be fully understood. A mutually beneficial relationship between the psyllid and certain species of endosymbiont bacteria, including *Wolbachia* and *Sodalis* species, has been reported (Cooper et al., 2022). In one scenario, *C*Lso could exist in the psyllid under a symbiotic relationship. In this context, *C*Lso may gain nutrition from the psyllid (Tsuchida et al., 2004), while the bacteria may offer psyllids protection from their natural predators, host plant defenses, insecticides, and/or other environmental hazards (Qiu and Scholthof, 2000; Montllor et al., 2002; Heyworth and Ferrari, 2016; Doremus and Oliver, 2017). In contrast, in a second scenario, *C*Lso could have a negative relationship as a pathogen/parasite of the insect. There are few reports of *C*Lso adversely affecting metabolic and reproductive fitness in *B. cockerelli* (Nachappa et al., 2014; Albuquerque Tomilhero Frias et al., 2020). The presence of *C*Lso in the psyllid reduces the efficacy of female oviposition and reproduction, which subsequently determines the selection of the host plants. The phenomenon is described as the preference–performance hypothesis (PPH) to ensure the successive transmission of the bacterium mostly in the vasculature tissue of the host plants (Mayhew, 1997; Gripenberg et al., 2010; Jones, 2022). Regardless, understanding the intricate interactions (beneficial or antagonistic) between the psyllid and the bacterium is crucial for developing strategies to control the psyllid- and *C*Lso-induced crop losses.

The fastidious (unculturable) nature of the *C*Lso bacterium thriving in limited environments within the insect and host plant tissues further hampers our understanding of the processes involved in pathogenesis. With the advent of next-generation sequencing, genome-wide studies have become a vital approach to studying the relationship between plant and insect vectors and the associated microbes. A few studies analyzed transcriptomes of *B. cockerelli* in response to *C*Lso by employing *de-novo* assemblies since the reference genome was previously unavailable (Nachappa et al., 2012; Huot et al., 2018). The results showed dynamic expression of psyllid genes and enrichment of functional terms related to the immune response under the influence of *C*Lso (Nachappa et al., 2012; Huot et al., 2018). We have also examined *C*Lso transcriptomes and identified potential candidate genes underlying plant-pathogen and psyllid interactions (Ibanez et al., 2014). However, tissue-specific expression profiles of the *B. cockerelli* vectoring *C*Lso remain unexplored.

In this study, leveraging the recently released psyllid reference genome (Kwak et al., 2023), we performed RNA-Seq and transcriptome analysis of the psyllid organs (salivary glands and ovaries), which are critical for *C*Lso biology and transmission. We identified differentially expressed genes (DEGs) under the influence of the *C*Lso bacterium, organ-specific genes, and co-expressed modules distinguishing the two distinct psyllid organs using weighted gene co-expression network analysis (WGCNA). Furthermore, comparative GO enrichment analysis revealed specific biological processes enriched among the ovaries and the salivary glands, implicating insights into *C*Lso acquisition, replication, and transmission in the psyllid vector.

## Materials and methods

### Insect maintenance, RNA isolation, and RNA-Sequencing

*B. cockerelli* colonies free of *C*Lso [*C*Lso(-)] or carrying *C*Lso (haplotype B) [*C*Lso(+)] were reared on potato plants and maintained at the Texas A&M AgriLife Research & Extension Center, Weslaco, Texas. The absence [*C*Lso(-)] or presence [*C*Lso(+)] of the *C*Lso bacterium in the psyllid salivary glands and ovaries was determined by employing molecular diagnostics; polymerase chain reaction (PCR) using 16S rDNA primers specific to the *C*Lso (Avila et al., 2019). Further, salivary glands and ovaries of teneral adult psyllids were carefully dissected under the microscope. Briefly, total RNA from three biological replicates representing the salivary glands and ovaries of *C*Lso(-) and *C*Lso(+) psyllids were extracted using a Direct-zol RNA Miniprep kit (Zymo Research, Irvine, CA). The quantity and quality of the RNA samples were estimated by NanoDrop-1000 spectrophotometer (Thermo Fisher Scientific, Waltham, MA) and Bioanalyzer (Agilent Technologies, Santa Clara, CA), respectively. The four RNA samples representing *C*Lso(-) and *C*Lso(+) conditions of the salivary glands and ovaries were further processed to prepare libraries and sequenced in paired-end mode to obtain 150 bp long reads per Illumina’s recommendations (San Diego, CA) at Texas A&M AgriLife Genomics and Bioinformatics Services (College Station, TX). The raw files of RNA-Seq data of the four samples, each with three biological replicates, have been deposited at NCBI’s Gene Expression Omnibus (GEO) under the PRJNA1076531 BioProject accession number.

### RNA-Seq data processing and gene expression analysis

The quality of the paired-end reads was analyzed using the NGS QC Toolkit using the default parameters (Patel and Jain, 2012). The high-quality reads were mapped to the reference genome of *B. cockerelli* (Kwak et al., 2023) using the STAR aligner at default settings (Dobin et al., 2013). The number of reads in each gene was estimated using the reference annotations (Kwak et al., 2023) and the feature Counts tool at the default settings (Liao et al., 2014). Differentially expressed genes (DEGs) under *C*Lso(+)/*C*Lso(-) [*C*Lso(-) as reference] in each organ and/or between the two organs (ovaries/salivary glands; salivary glands as reference) under *C*Lso(+) and *C*Lso(-) conditions were estimated with the default normalization algorithms implements in DESeq2 tool (Love et al., 2014). In addition, each gene’s expression level was estimated using the normalized read count data by considering read depth and gene size (fragments per kilobase per million mapped fragments; FPKM).

### Principal component analysis

Principal component analysis (PCA) was performed using the expression values (FPKM) with the Factoextra package in the R program to examine the relationship among the four samples. The Pearson correlation coefficient (R) among the four samples was estimated and shown via heatmap using Python scripts.

### Chromosome-wide gene expression and distribution

To examine the chromosome-wide expression patterns, we first classified all genes into expressed (≥0.5 FPKM) or not-expressed (<0.5 FPKM) categories. Subsequently, the frequency of expressed genes per 100 KB was estimated and plotted via Circos-plot (Krzywinski et al., 2009). Likewise, the chromosome-wide expression level was estimated and visualized using box plots.

### Tissue-specific expression analysis

To examine organ-specific expression under *C*Lso(+) and *C*Lso(-) conditions, we estimated the tissue specificity index (TSI) (Kryuchkova-Mostacci and Robinson-Rechavi, 2017) among the four samples. Only the expressed (≥0.5 FPKM) genes in any of the four samples were selected and used as input to determine TSI in the R program. Those genes with ≥0.9 TSI in any of the four samples were considered specifically expressed, as described previously (Jain et al., 2022a).

### Gene co-expression network analysis

We performed a weighted gene co-expression network (WGCNA; v1.61) (Langfelder and Horvath, 2008) analysis among the four distinct samples to identify sets of co-expressed genes exhibiting significant correlation. Genes with ≥0.1 FPKM in any of the four samples were selected and transformed into a log_2_ scale. Those genes with ≥0.1 variances (based on their log_2_ transformed FPKM values) were further used to determine significantly correlated modules. A cut-off of 9 *β*-value (soft-threshold power) and scale-free topology index (R^2^) was used to estimate the adjacency matrix and sets of co-expressed modules with high correlation (R) and significance (*P*-value) as described earlier (Garg et al., 2017). The relationship of the co-expressed modules was determined using the default parameters implemented in the WGCNA program and displayed as a dendrogram.

### Gene ontology analysis

To examine the functional relevance of the DEGs and co-expressed genes, we performed gene ontology (GO) enrichment analysis using the BINGO plug-in implemented in Cytoscape (v3.9.1) (Shannon et al., 2003). The enriched GO terms with <0.05 *P*-value were considered significant. The number of genes and significance level (*P*-value) in each enriched GO term were presented via bubble plots generated in the R program. Moreover, comparative GO analyses among the different sets of genes were examined using the EnrichmentMap tool implemented in Cytoscape (v3.9.1) (Shannon et al., 2003).

### Identification of genes encoding transcription factors

To assign transcription factors (TFs), we performed a local blastP between amino acid sequences of the psyllid proteins with amino acid sequences of *Drosophila melanogaster* TFs available in AnimalTFDB (v3.0) (Shen et al., 2023). The most significant match with a cutoff of <0.005 *P*-value was assigned for each gene of *D. melanogaster* to determine TFs in *B. cockerelli*.

## Results and discussion

### Genomic and transcriptomic attributes of *B. cockerelli*

Psyllid tissue-specific transcriptome rendered in a total of 8.44-11.78 million paired-end reads for each sample. Of these, 93-95% were high-quality reads, as determined by the NGS QC Toolkit (Patel and Jain, 2012). The reads were next mapped to the *B. cockerelli* reference genome (Kwak et al., 2023). The current *B. cockerelli* genome draft is the first release and consists of ~4409 scaffolds of at least >1 kb size, with 13 biggest scaffolds representing 13 distinct chromosomes, covering ~92% of the psyllid genome (Kwak et al., 2023). Due to noncontiguous sequences in the smaller scaffolds, we only mapped the RNA-Seq reads to the 13 biggest scaffolds. In total, ~4.71-7.48 million read pairs were mapped uniquely (~53-66%) to the 13 biggest scaffolds of the reference genome (Kwak et al., 2023) (**Supplementary Table 1**). The mapping percentage could be marginally higher if the reads were mapped to all other scaffolds. Approximately 19,699 genes were annotated on the 13 chromosomes (Kwak et al., 2023). All the downstream transcriptome analyses were performed with these reference gene annotations.

**Figure 1:**
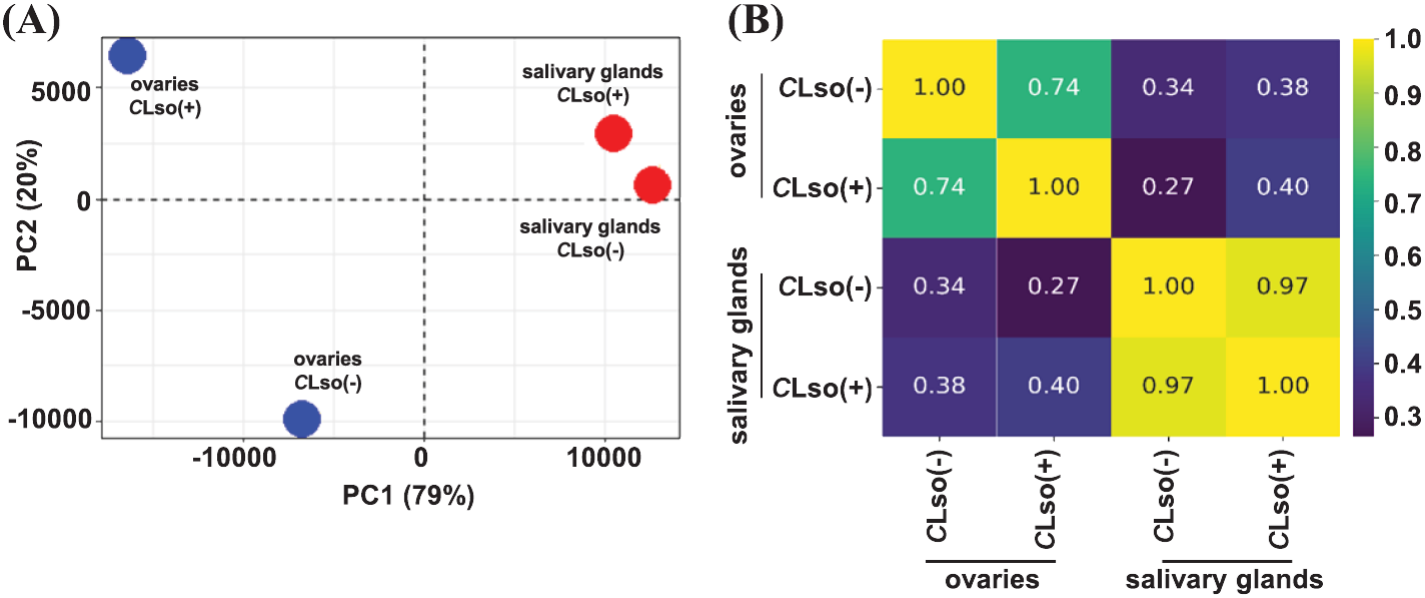
Transcriptome profile of the tomato-potato psyllid organs in response to *C*Lso infection. **(A, B)** Correlation among transcriptomes of the psyllid salivary glands and ovaries in control psyllids [*C*Lso(-)] and those carrying bacterium [*C*Lso(+)] is shown via PCA plot **(A)** and heatmap **(B)**. The scale depicts Pearson’s correlation coefficient (R).

First, we examined the broader relationships of the four distinct samples representing the salivary glands and ovaries of *C*Lso(-) and *C*Lso(+) psyllids using the FPKM expression metric. The transcriptome profiles of the salivary glands and ovaries were quite distinct, with Pearson’s correlation of 0.34 and 0.38 under *C*Lso(-) and *C*Lso(+) conditions, respectively. In response to *C*Lso [*C*Lso(-) vs *C*Lso(+)] within the specific organ(s), a correlation of 0.97 and 0.74 were detected in the salivary glands and ovaries, respectively (**Figures 1A, B**). These correlations were as expected since transcriptomes of distinct tissue types/organs would be more disparate than the overall gene expression profiles influenced by CLso’s presence. Recently, a tissue-specific transcriptome analysis of another closely related psyllid, *Diaphorina citri* (Asian citrus psyllid), vectoring *Candidatus* Liberibacter asiaticus (*C*Las), the presumptive causal agent of Huanglongbing disease was reported (Mann et al., 2022). The results showed unique transcriptome profiles in the distinct organs responding to *C*Las (Mann et al., 2022). Our results in *B. cockerelli* in response to *C*Lso were broadly similar to those observed for the tissue-specific transcriptomes of the Asian citrus psyllid vectoring *C*Las in the virtue of organ-specific transcriptome signatures under the influence of fastidious bacteria (Mann et al., 2022).

### Chromosome-wide transcriptome dynamics of *B. cockerelli*

The 13 chromosomes of *B. cockerelli* were assigned into 12 autosomes and one sex chromosome (Kwak et al., 2023). The size of the autosomes ranged from 23.61 65.45 Mb, while the sex chromosome spanned 22 Mb (Kwak et al., 2023). The genomic attributes of the ~19,699 *B. cockerelli* annotated genes and their expression dynamics remain unexplored. Here, we analyzed the length distribution of the ~19,699 *B. cockerelli* genes. A substantial fraction (26.3%) of the genes were ∼1-3 kb long (**Supplementary Figure 1A**). Next, we examined the chromosome-wide expression profiles based on the FPKM metric. Genes with ≥0.5 FPKM were considered expressed (Jain et al., 2022b). Among the 12 autosomes, ~980-2888 genes were located per chromosome. Of them, ~535-1,598 (~37.96-61.2%) genes were found to be expressed in any of the four distinct samples analyzed. A similar analysis was performed with the single sex chromosome, too. The percentage of expressed genes on the sex chromosome was greater (58.59-68.98%; 531 out of 693 annotated genes) than the autosomes (**Supplementary Figure 1B**; **Supplementary Table 2**). Further, we estimated the frequency of expressed genes in a window size of 100 kb, as shown via Circos-plot. In general, the expressed genes were found to be evenly distributed across the length of most of the chromosomes (**Supplementary Figure 2A**). Interestingly, ChrX harbored the highest frequency (58.59-68.98%) of expressed genes (≥0.5 FPKM) and the least frequency in Chr12 (37.96-44.59%) and Chr1 (39.44-43.39%) in any of the four samples analyzed (**Supplementary Figures 1B**, **2A**). Moreover, the expression level of genes located in ChrX (1.18-2.69; log_2_ transformed FPKM median expression) also exhibited higher expression than autosomes (0.16-0.63; log_2_ transformed FPKM median expression) in all four samples analyzed (**Supplementary Figure 2B**). The high-level expression of genes on the sex chromosome may be critical for regulating biological processes related to sex determination and governing reproductive development processes in *B. cockerelli*.

**Figure 2:**
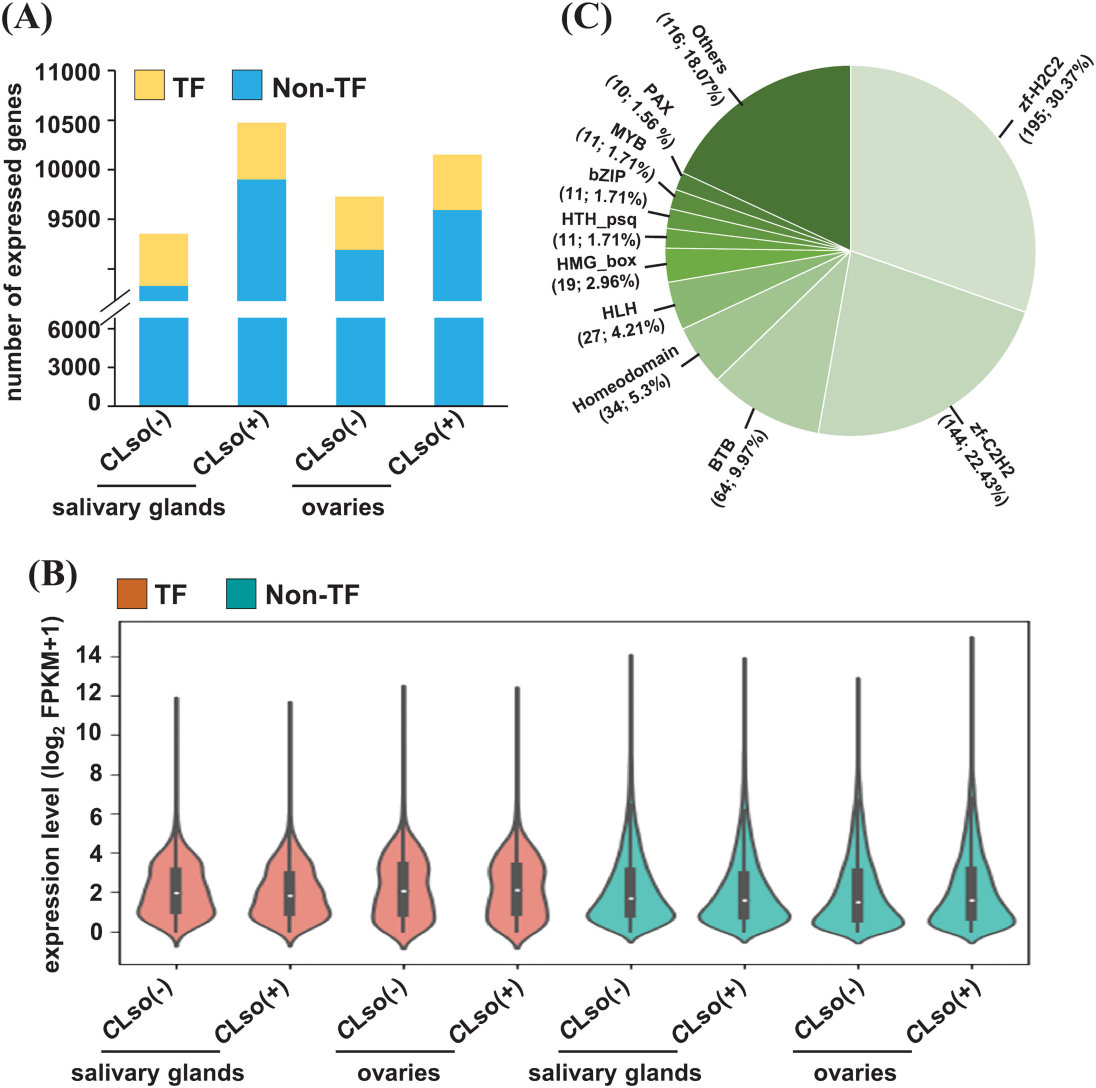
Expression profile of genes encoding TFs and non-TFs. **(A)** The number of expressed (≥0.5 FPKM) genes encoding TFs and non-TFs in the salivary glands and ovaries in control psyllids [*C*Lso(-)] and those carrying bacterium [*C*Lso(+)] is shown in a bar plot. **(B)** The expression level of the genes encoding TFs and non-TFs of the four samples given in **(A)** is shown via a violin plot. **(C)** The fraction of different types of expressed TFs in any of the four samples given in **(A, B)** is shown via a pie chart.

Transcription factors (TFs) play an important role in regulating the expression of downstream genes via binding in their cis-regulatory elements (CREs) (Priest et al., 2009; Wittkopp and Kalay, 2011). However, the genes encoding TFs in *B. cockerelli* are not well annotated or described in the released genome (Kwak et al., 2023). Therefore, we annotated and characterized the TFs in *B. cockerelli* based on the most significant match with drosophila’s TFs available in AnimalTFDB (v3.0) (Shen et al., 2023). A total of 801 genes encoding TFs were detected in *B. cockerelli*. Of these, 528-575 TFs were found to be expressed with ≥0.5 FPKM (**Figure 2A**). The percentage of expressed TFs (65.92-71.79%) was substantially higher than non-TF genes (46.71-52.39%) across the four different samples (**Figure 2B**; **Supplementary Table 3**). Among the expressed TFs, zf-H2C2 (30.37%), zf-C2H2 (22.43%), BTB (9.97%), homeodomain (5.3%), and HLH (4.21%) were the five topmost represented classes of TFs (**Figure 2C**; **Supplementary Table 3**).

**Figure 3:**
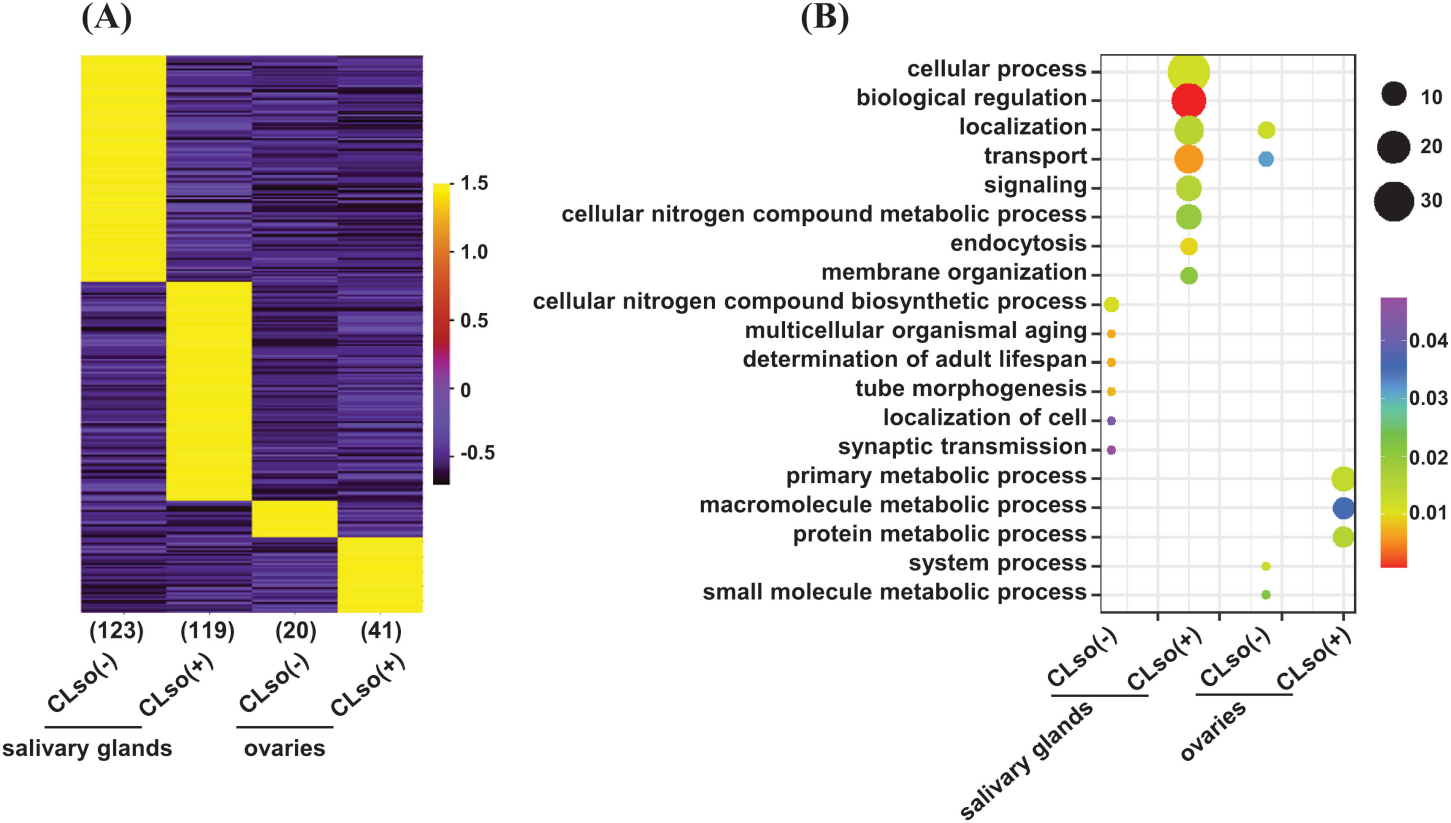
A tissue-specific expression of the psyllid genes in response to *C*Lso. **(A)** Heatmap showing the expression level of genes expressed in a tissue-specific manner (≥0.9 TSI) of the psyllid salivary glands and ovaries under *C*Lso(-) and *C*Lso(+) conditions. The scale represents the expression level (FPKM) in a row-wise z-score. The number of genes in each category is shown at the bottom of the heatmap. **(B)** The enriched GO terms in the uniquely expressed genes in the salivary glands and ovaries under *C*Lso(-) and *C*Lso(+) conditions are shown via a bubble plot. The scale represents the significance level (*P*-value) and number of genes in each enriched GO term.

Further, to examine genes expressed in an organ-specific manner in the salivary glands and ovaries in the *C*Lso(-) or *C*Lso(+) conditions, we estimated the tissue specificity index (TSI) of the four distinct samples using their FPKM values (Kryuchkova-Mostacci and Robinson-Rechavi, 2017). The relevance and estimation methods are quite different from differential expression analysis. In TSI, a set of uniquely expressed genes is determined in a tissue/organ/condition-specific manner with reference to all the other remaining samples. In contrast, differential expression analysis is performed between two samples, representing only one reference. TSI analysis showed that 123 and 20 genes exhibited tissue-specific expression under the *C*Lso(-) condition in the salivary glands and ovaries, respectively. Likewise, 119 and 41 genes showed a tissue-specific expression under the *C*Lso(+) condition in the salivary glands and ovaries, respectively (**Figure 3A**). Gene ontology (GO) analysis revealed significant enrichment of processes involved in localization/transport, signaling, and endocytosis preferentially/specifically under *C*Lso(+) condition in the salivary glands. Likewise, specific enrichment of GO terms involved in the primary metabolic process, macromolecule metabolic process, and protein metabolic processes were observed under the *C*Lso(+) condition in the ovaries. In contrast, significantly enriched processes under the *C*Lso(-) condition in both organs were represented with only a few genes, rendering only a trivial correlation in the absence of *C*Lso (**Figure 3B**). In addition, other attributes of GO enrichment analysis, such as molecular function and cellular component, were interrogated. Molecular function GO terms involved in DNA binding, signal transducer activity, ATP binding, and membrane transporter activity were enriched in specifically expressed genes in salivary glands under *C*Lso(+) condition, and localized in the membrane, plasma membrane, membrane-bound vesicles (**Supplementary Figures 3A, B**). Likewise, GO terms related to catalytic activity, peptidase/endopeptidase activity, and localization in intracellular spaces were enriched under the *C*Lso(+) condition in ovaries. Conversely, the enrichment of the molecular function and cellular component GO terms were underrepresented in *C*Lso(-) condition in both the salivary glands and ovaries (**Supplementary Figures 3A, B**). We suggest that the influence of *C*Lso might be implicated in determining unique signatures of the two distinct organs via regulating stress-responsive biological processes and functions.

### Differential expression analysis in response to *C*Lso within the distinct organ(s)

**Figure 4:**
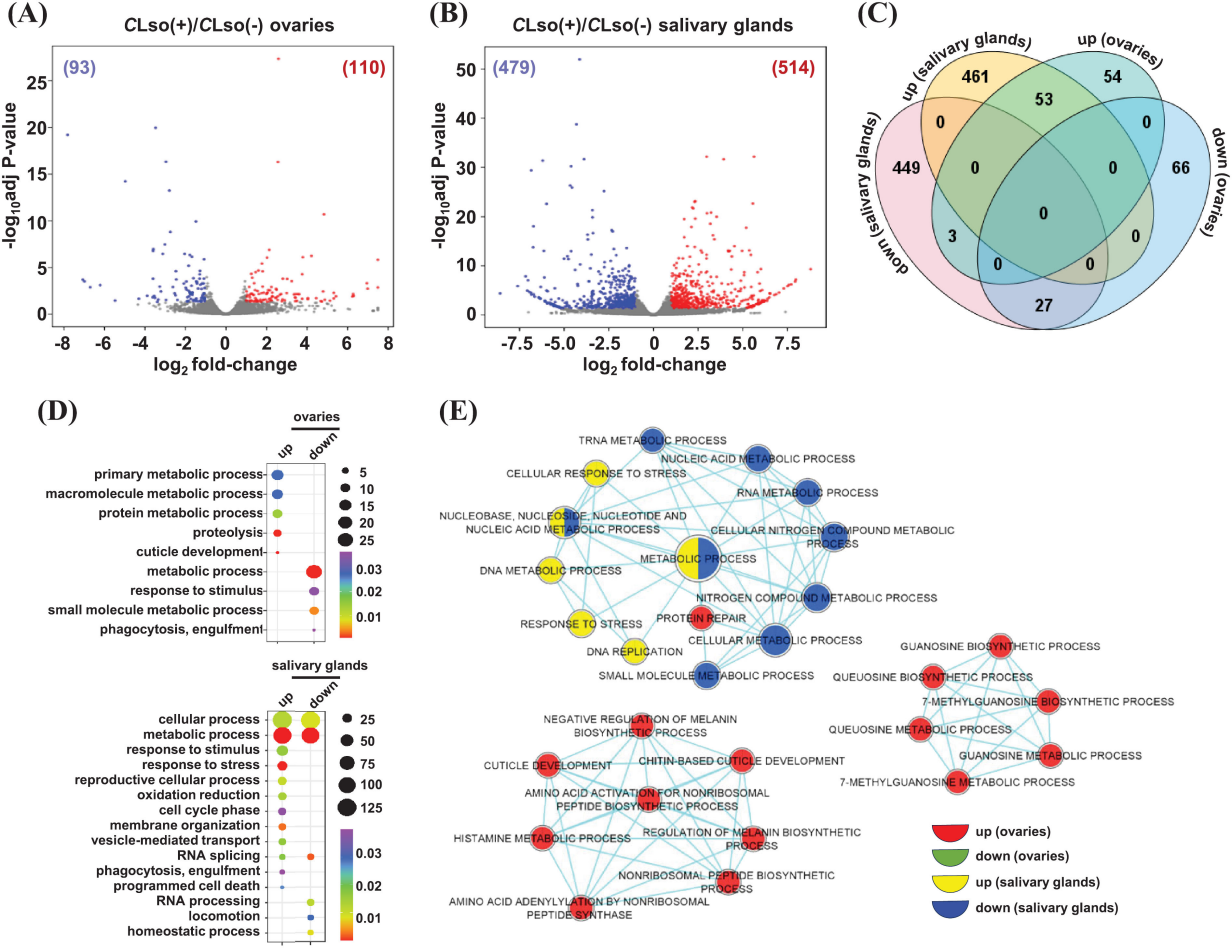
Differential expression in response to *C*Lso in the tomato-potato psyllid organs. **(A, B)** The differential expression profiles in response to *C*Lso as compared to the uninfected control [*C*Lso(+)/*C*Lso(-)] in the ovaries **(A)** and salivary glands **(B)** are shown via volcano plots. The number of up- and down-regulated genes is given in parentheses. **(C)** The number of genes exhibiting common and unique among the up- and downregulated genes given in **(A, B)** is shown via Venn diagram. **(D)** The enrichment of GO terms for the sets of uniquely up and downregulated genes in response to *C*Lso in the two distinct organs are shown. Scales represent the significance level (*p*-value) and number of genes in each enriched GO term. Different colors indicated the enrichment of GO terms in each class of genes. **(E)** A comparative enrichment of GO terms across the four sets of uniquely up and downregulated genes in response to *C*Lso in the two distinct organs. Node size indicates the number of genes, and different colors depict different sets of DEGs.

A few previous studies analyzed the differential expression of genes in response to *C*Lso in the whole psyllid (*B. cockerelli*) (Nachappa et al., 2012; Huot et al., 2018). The psyllid salivary glands and ovaries are vital organs for pathogenesis and transmission to the host plants. Therefore, a tissue-specific differential expression analysis in these organs in response to *C*Lso may provide blueprints for insect-pathogen interactions. We identified DEGs between *C*Lso(-) and *C*Lso(+) conditions [*C*Lso(-) as reference] within the distinct organ(s). A total of 110 and 93 genes exhibited up- and downregulation under the *C*Lso(+)/*C*Lso(-) condition in the ovaries (**Figure 4A**; **Supplementary Table 4**). Likewise, 514 and 479 genes showed up- and downregulation in response to *C*Lso in the salivary glands, respectively (**Figure 4B**; **Supplementary Table 4**).

Next, we identified common and unique sets of genes that were up- and downregulated in response to *C*Lso in the two psyllid organs. A total of 54 and 461 genes were upregulated explicitly in the ovaries and salivary glands, respectively. Likewise, 66 and 449 genes exhibited specific downregulation in the ovaries and salivary glands, respectively (**Figure 4C**). To examine the functional relevance of these uniquely up- and downregulations in response to *C*Lso, we analyzed the enrichment of GO terms. Our results showed that specifically upregulated genes in *C*Lso-infected ovaries were related to GO terms involved in primary/macromolecule/protein metabolic processes, proteolysis, and cuticle development. For instance, the GO term ‘proteolysis’ contained the genes encoding *trypsin 1*, *carboxypeptidase N*, and *cathepsin L1*. These are critical proteolytic enzymes involved in diverse biological processes, such as reproduction, embryo development, and immunity (Law et al., 1977; Krautz et al., 2014; Jagdale et al., 2017; Yu et al., 2019; Ferrara et al., 2020). The upregulation of *cathepsin L1* (*BcCathL1*) in psyllid ovaries might suggest that the host is mounting a tissue-specific immune response against *C*Lso. Also, it could be associated with ovarian diapause, reducing the number of eggs oviposited, as was demonstrated in *Coccinella septempunctata* (Linnaeus) (Chen et al., 2022). The transcriptional modulation of proteolytic enzymes (upregulation) determined in our study might also be related to a previous study by Albuquerque Tomilhero Frias et al., 2020, wherein *C*Lso-infected females (haplotype B, same as used in this study) showed reduced oocyte development and eggs oviposited compared to uninfected psyllids. We hypothesize that reduced progeny could be associated with the survival and reproduction of the *C*Lso-infected psyllids in the host plants. A body of evidence revealed the preference–performance hypothesis (PPH), which determines the choice of host plant selection by the phytophagous insect to ensure female oviposition and successive transmission of the offspring (Mayhew, 1997; Gripenberg et al., 2010). The phenomenon of PPH is supposedly implicated in the *C*Lso-infected phytophagous psyllids for their successive transmission. Likewise, GO terms related to cellular/metabolic processes, response to stimuli/stress, reproductive cellular process, oxidation-reduction, cell cycle phase, membrane organization, vesicle-mediated transport, RNA splicing, phagocytosis, and programmed cell death were enriched in specifically upregulated genes in the salivary glands (**Figure 4D**). For instance, transcripts of *Carboxypeptidases* (E and M) from the GO term ‘vesicle-mediated transport’ were up-regulated explicitly in *C*Lso-infected salivary glands. Carboxypeptidases belong to a large family of enzymes governing the cleavage of C-terminal residues (Gomis-Rüth, 2008) and they play an important role in several biological processes, such as control of peptide activity at the cell surface and post-translational processing of extracellular proteins and peptides (Reznik and Fricker, 2001). The function of Carboxypeptidases is still unclear in tomato/potato-psyllid-*C*Lso interactions. However, these proteins could be plausibly involved in the defense response of the host plants. We hypothesize that the colonization of salivary glands by *C*Lso may modulate the expression of host vesicle-mediated transport-related genes to evade plant-immune defenses towards the pathogen, degrading critical proteins and peptides necessary to reduce insect feeding behavior and the probability of *C*Lso transmission. An electrical penetration graph study on CLso-infected psyllids showed a significant increase in salivation, phloem ingestion, and several probes in comparison to the uninfected psyllids (Valenzuela et al., 2020), supporting our hypothesis; further investigations will unveil the function of vesicle-mediated transport-related genes in the potato psyllid-*C*Lso interaction. Conversely, GO terms involved in the metabolic process, response to stimuli, small molecule metabolic process, and phagocytosis were enriched in the specifically downregulated genes in the ovaries. Likewise, GO terms involved in RNA splicing/processing, locomotion, and homeostatic processes were enriched in specifically downregulated genes in the salivary glands (**Figure 4D**).

Next, we performed a comparative GO among the four sets of specifically up- and downregulated genes in response to *C*Lso. The comparative GO enrichment analysis enables us to identify relatively more significant GO terms among the multiple sets of genes with greater stringent criteria than the conventional GO enrichment analysis from a single set of genes. Amino acid/histamine/guanosine metabolic processes, cuticle development, and protein repair terms were exclusively enriched in specifically upregulated genes in the ovaries. Likewise, in response to stress, DNA metabolic and DNA replication-related GO terms were enriched in specifically upregulated genes in the salivary glands (**Figure 4E**). Conversely, no GO terms were enriched in specifically downregulated genes in the ovaries. However, metabolic processes related to nucleic acid/nitrogen compound/small molecule were enriched in specifically downregulated genes in the salivary glands (**Figure 4E**). Further analysis of the molecular function and cellular component GO terms revealed the enrichment of metal ion binding, hydrolase activity, peptidase activity, and localization in intracellular spaces in the upregulated genes in response to the presence of *C*Lso bacterium in ovaries. Likewise, GO terms involved in metal ion binding, hydrolase activity, oxidoreductase activity, ATPase binding, GTP binding, and helicase activity were preferentially enriched and localized in intracellular spaces, cytoplasm, cytoskeleton, and ribonucleoprotein complexes in the presence of *C*Lso in the salivary glands (**Supplementary Figures 4A, B**). Conversely, enriched GO terms in downregulated genes in the presence of *C*Lso in the ovaries included metal ion binding and oxidoreductase activity. Likewise, cofactor binding, transferase activity, lyase activity, nuclease activity, UDP-glucosyltransferase activity, and localization into the cytoskeleton, nucleus, microtubule-associated complex, and lipid particle were identified in the set of downregulated genes in the presence of *C*Lso in the salivary glands (**Supplementary Figures 4A, B**). Overall, the results suggest that distinct transcriptional changes in multiple biological processes and functions in salivary glands and ovaries are crucial for the *C*Lso-potato/tomato psyllid interactions.

### Transcriptome dynamics across the distinct organs in *B. cockerelli*

**Figure 5:**
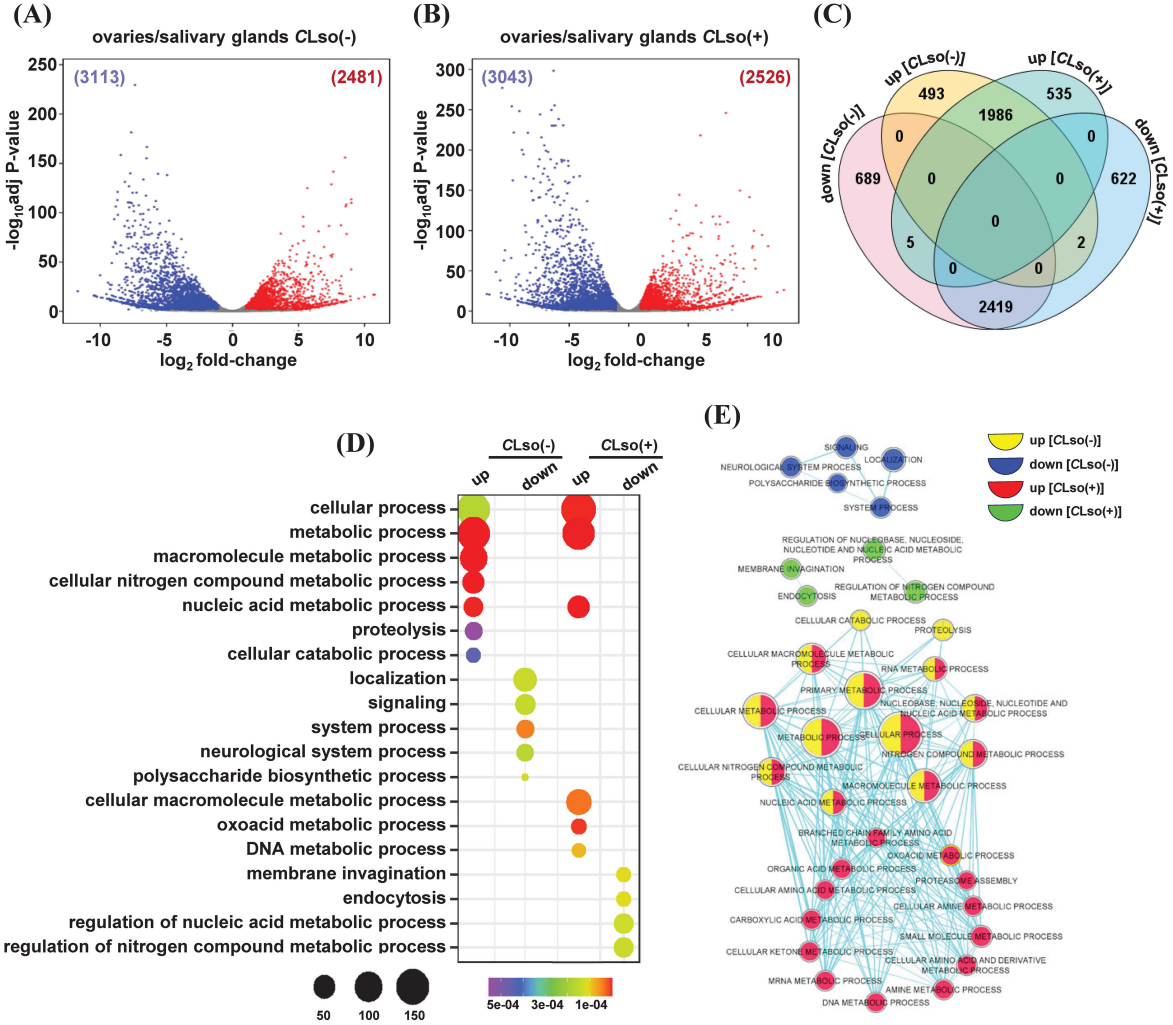
Differentially expressed genes between the ovaries and salivary glands in tomato-potato psyllid. **(A, B)** Differential expression profiles between the ovaries and salivary glands in control [*C*Lso(-)] and those carrying bacterium [*C*Lso(+)] **(B)** are shown via volcano plots. The number of up- and down-regulated genes is given in parentheses. **(C)** The number of genes exhibiting common and unique among the four sets of up- and downregulated genes given in **(A, B)** is shown via Venn diagram **(D)**. The enrichment of GO terms of the uniquely up- and downregulated genes given in **(C)** is shown via a bubble plot. Scales represent the significance level (*P*-value) and number of genes in each enriched GO term. **(E)** A comparative GO enrichment among the four sets of uniquely up- and downregulated given in **(C)** is depicted with a network. Different colors indicated the enrichment of GO terms in each class of genes.

After initial acquisition by *B. cockerelli, C*Lso multiplies and circulates in the body of the psyllid. About two weeks after the acquisition, the *C*Lso reaches the salivary glands and acquires potency to infect the host plants (Sengoda et al., 2013; Rashed et al., 2014; Sengoda et al., 2014). In contrast, replication and vertical transmission of *C*Lso in successive generations are mostly confined to the ovaries and eggs of the psyllid (Hansen et al., 2008; Casteel et al., 2012; Kean et al., 2019). Therefore, we next performed a comparative analysis between the two organs (ovaries/salivary glands) in the presence or absence of the bacterium. In the control psyllids [*C*Lso(-)], a total of 2,481 and 3,113 genes exhibited up- and downregulations, respectively (**Figure 5A**; **Supplementary Table 5**). Likewise, 2,526 and 3,043 genes showed up- and downregulations, respectively, under the *C*Lso(+) condition (**Figure 5B**; **Supplementary Table 5**). Further, these four sets of up- and downregulated genes were used to identify uniquely up- and downregulated genes. In total, 493 and 689 genes exhibited specific up- and downregulation between the ovaries and salivary glands under the *C*Lso(-) condition. Likewise, 535 and 622 genes showed exclusively up- and downregulation, respectively, under the *C*Lso(+) condition (**Figure 5C**).

We performed an enrichment analysis of GO terms to gain insights into the functional relevance of these specifically up- and downregulated genes. Under the *C*Lso(-) condition and upregulation in the ovaries, GO terms involved in macromolecule/nitrogen compound/nucleic acid metabolic processes, cellular catabolic process, and proteolysis were enriched. Conversely, GO terms involved in localization, signaling, neurological system process, and polysaccharide biosynthetic process were enriched under *C*Lso(-) condition and downregulated in the ovaries (**Figure 5D**). Likewise, in response to *C*Lso(+) condition and upregulation in the ovaries, GO terms involved in nucleic acid/cellular macromolecule/DNA metabolic processes were enriched. However, GO terms related to nucleic acid/nitrogen compound metabolic processes, membrane invagination, and endocytosis were enriched in the set of downregulated genes under *C*Lso(+) condition (**Figure 5D**).

Further, we performed comparative GO analysis among the specifically up- and downregulated genes between the ovaries and salivary glands under the *C*Lso(-) and *C*Lso(+) conditions. Metabolic processes related to nucleic acid/RNA/nitrogen compound/macromolecule were enriched in the upregulated genes in the ovaries under both the *C*Lso(-) and *C*Lso(+) conditions. However, metabolic processes involved in amino acid/carboxylic acid/ketone bodies/organic acid were exclusively enriched in response to *C*Lso(+) condition in the ovaries. Conversely, processes involved in membrane invagination and endocytosis were specifically enriched in the set of downregulated genes under the *C*Lso(+) condition (**Figure 5E**). Previous studies showed that the reproductive fitness of *Bactericera cockerelli* is adversely affected by *C*Lso’s presence (Nachappa et al., 2014; Albuquerque Tomilhero Frias et al., 2020). Such antagonistic relationships have been studied earlier (Schwenke et al., 2016). The *C*Lso replication and psyllid reproduction processes are physiologically and energetically demanding and governed by insect hormones, 20-hydroxyecdysone, and insulin/insulin-like growth factor via endocrine-regulated energy metabolism pathway (Lorenz and Gerd Gäde, 2009; Schwenke et al., 2016). Our results suggest that the perturbation of various metabolic processes in response to *C*Lso in the ovaries could be corroborated by a trade-off between lower reproductive fitness and enhanced immunity of the psyllids. Further analysis of the molecular function and cellular component GO terms revealed enrichment of hydrolase activity, nucleotide binding, and transferase/methyltransferase activity and localization into membrane-bound organelle, nucleus, nuclear lumen, and nucleoplasm in the set of upregulated genes in ovaries in the presence of *C*Lso bacterium. Likewise, nucleotide binding, transferase activity, ATP binding, and hydrolase/helicase/ligase activity and localization into the membrane and membrane-bound organelle were identified among the upregulated genes in the ovaries in the absence of *C*Lso bacterium (**Supplementary Figures 5A, B**). Conversely, the enriched GO terms in downregulated genes were minimal in the presence of *C*Lso while a few GO terms, such as cation binding, transcription regulator activity, and mono-oxygenase activity and, localization into the membrane-bound organelle, and membrane were enriched in the absence of the *C*Lso bacterium (**Supplementary Figures 5A, B**).

Next, we analyzed the differential expression of genes encoding TFs between the ovaries and salivary glands under *C*Lso(-) and *C*Lso(+) conditions. In total, 31 and 25 TFs under the *C*Lso(-) condition showed up- and downregulation in the ovaries, respectively. Likewise, a total of 19 and 23 TFs were specifically up- and downregulated, respectively, under *C*Lso(+) condition in the ovaries (**Supplementary Figure 6A**). Among them, TFs encoding zinc-finger (zf-H2C2 and zf-C2H2) types, BTB, homeodomain, HMG box, HLH, and bZIP were predominant (**Supplementary Figure 6B**). Zinc finger and bZIP proteins are involved in diverse processes, including growth, development, and immune response in different organisms (Alves et al., 2013; Fu and Blackshear, 2017; Rakhra and Rakhra, 2021; Zheng et al., 2021). Likewise, HLH and HMG TFs were implicated in defense responses (Malarkey and Churchill, 2012; Lee et al., 2014; Chen et al., 2017; Cook et al., 2020). The TFs perturbed in the psyllid organs and associated with *C*Lso could also mediate signal transduction and contribute to psyllid growth, development, and stress responses. TFs play a crucial role in regulating the expression of several associated downstream genes and their dynamic expression between the ovaries and salivary glands of the psyllids in the absence or presence of *C*Lso bacterium may provide blueprints and mechanistic insights into the pathogenesis and transmission of the diseases to the host plants.

### Co-expression analysis revealed distinct modules governing Psyllid and *C*Lso interactions

**Figure 6:**
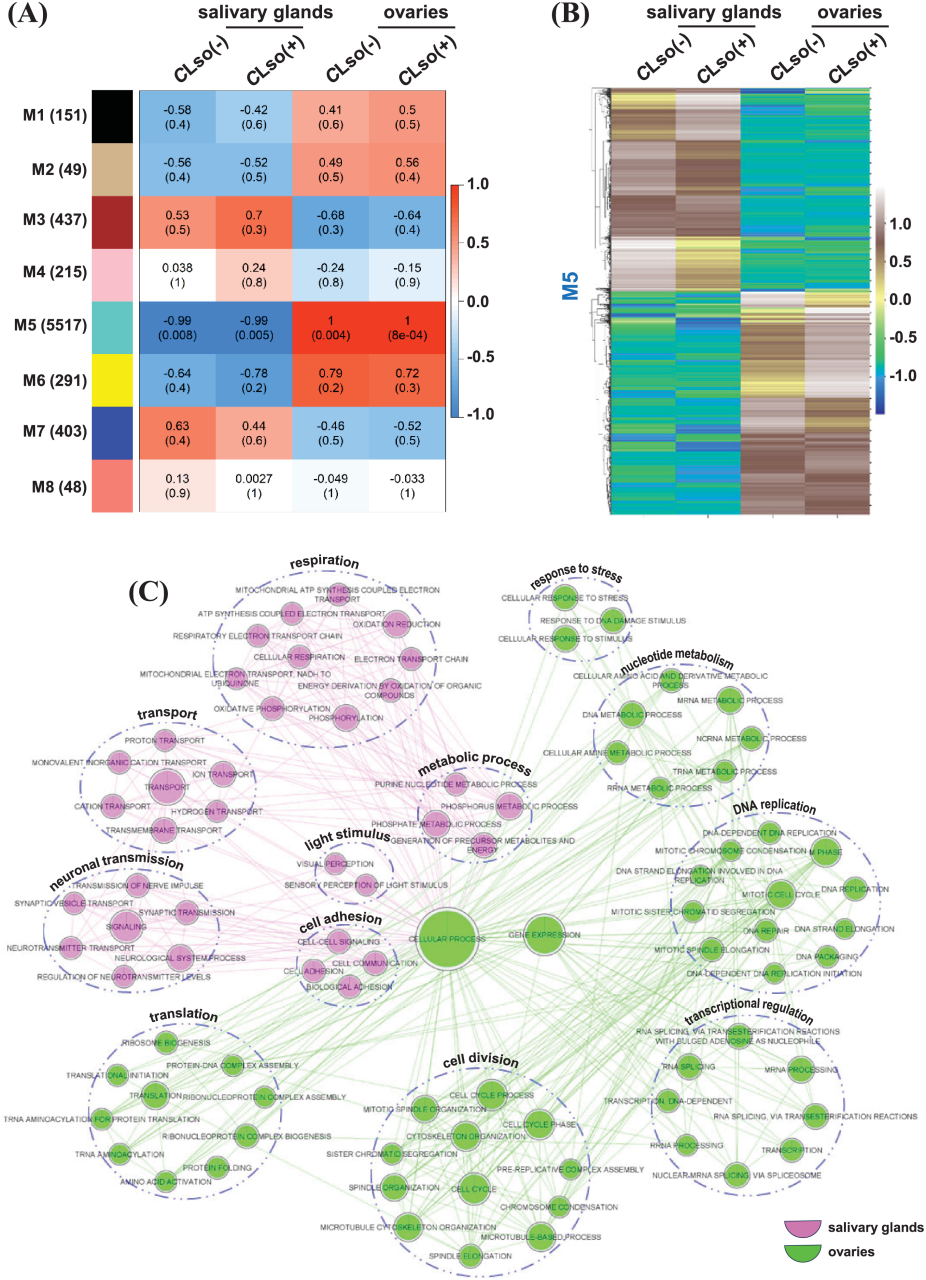
Co-expressed genes distinguishing tomato-potato psyllid salivary glands and ovaries. **(A)** Different sets (modules) of co-expressed genes as determined by correlation (R) and level of significance (*P*-value) among the four samples representing the psyllid salivary glands and ovaries under *C*Lso(-) and *C*Lso(+) conditions are shown. The scale represents Pearson’s correlation coefficient (R). The values inside and outside the parenthesis depict the level of significance (*P*-value) and Pearson’s correlation coefficient (R) is estimated using the default parameters implemented in the WGCNA program. **(B)** The expression profile of the genes belonging to the M5 module differentiating psyllid salivary glands and ovaries under *C*Lso(-) and *C*Lso(+) conditions is shown via heatmap. The scale represents expression level (FPKM) in row-wise z-scores. **(C)** The differential enrichment of GO terms between the salivary glands and ovaries in a set of co-expressed genes belonging to the M5 module under the *C*Lso(-) and *C*Lso(+) conditions is shown.

Gene co-expression and regulatory networks allow a better understanding of biological functions and processes at a modular level and can uncover new insights (Iyer-Pascuzzi et al., 2011; Macneil and Walhout, 2011). We employed a weighted gene co-expression network (WGCNA) to identify modules of co-expressed genes. Eight modules (M1-M8) were identified, with the highest number of genes in the M5 module (5517). In addition, only the M5 module exhibited a significant (*P*-value ranging from 0.008 to 8e-04) correlation under *C*Lso(-) and *C*Lso(+) conditions in the salivary glands and ovaries (**Figure 6A**; **Supplementary Figure 7**). The pattern of modules was contrasting between the salivary glands and ovaries but largely similar between the *C*Lso(-) and *C*Lso(+) conditions within the individual organ(s). However, a subtle difference in the significance level (*P*-value) in the M5 module was observed between the *C*Lso(+) and *C*Lso(-) conditions. In the salivary glands, the significance level under *C*Lso(+) (*P*-value = 0.005) was slightly higher than the *C*Lso(-) condition (*P*-value = 0.008). Likewise, a lower significance level was observed under *C*Lso(+) (*P*-value = 8e-04) than the *C*Lso(-) condition (*P*-value = 0.004) in the ovaries. The correlation and significance level of the co-expressed genes between the *C*Lso(-) and *C*Lso(+) conditions of the M5 module were similar in both the salivary glands and ovaries. Conversely, a contrasting correlation and significance level of the co-expressed genes between the salivary glands (R=-0.99) and the ovaries (R=1.0) was observed (**Figures 6A, B**).

Next, we performed a comparative GO enrichment analysis to gain deeper insights into the role of preferentially co-expressed genes in the salivary glands and ovaries. Interestingly, transport, neuronal transmission, cell adhesion, light stimulus, and respiration processes were enriched in the set of genes preferentially expressed in the salivary glands (**Figure 6C**). Many of these processes are relevant to transmitting the bacterium to the vasculature of host plants. For instance, neuronal transmission in Drosophila controls immunity and governs behaviors to coexist with the pathogens (Montanari and Royet, 2021). Cell adhesion molecules facilitate the invasion and colonization of the pathogens in the host organism (Bisht and Meena, 2019). In addition, transport and respiration are physiologically and energetically demanding processes during the infection of the host organisms (Porcheron et al., 2013; Killiny et al., 2018). In agreement, previous studies in *B. cockerelli* have shown that salivary glands are critical for *C*Lso transmission to host plants (Sengoda et al., 2013; Rashed et al., 2014; Sengoda et al., 2014). Likewise, GO terms involved in DNA replication, transcriptional regulation, translation, cell division, and response to stress were enriched in the set of preferentially expressed genes in the ovaries (**Figure 6C**). The enrichment of processes related to bacterial replication, specifically in the ovaries, is concurrent with the previously reported vertical transmission of the bacterium in the eggs and ovaries of the psyllid (Hansen et al., 2008; Casteel et al., 2012; Kean et al., 2019). Further, we examined the enrichment of molecular function and cellular component GO terms in the distinct sets of genes preferentially expressed in the salivary glands and ovaries. In the salivary glands, GO terms involved in cation binding, transporter, oxidoreductase, substrate-specific channel, ATPase, cofactor binding, NADH dehydrogenase, voltage-gated channel, and calmodulin activity were enriched and localized in the cytoplasm, plasma membrane, organelle membrane, mitochondria, and respiratory chain (**Supplementary Figures 8A, B**). In contrast, the enrichment of GO terms in the set of ovaries’ preferential genes included hydrolase, nucleotide binding, ATP binding, transferase activity, pyrophosphatase, ligase, helicase, RNA polymerase, and chromatin binding function, and localization into the cytoplasm, cytoskeleton, nucleus, chromosome, ribosome, and proteasome complex were identified (**Supplementary Figures 8A, B**). Overall, co-expression network analysis identified salivary gland and ovary-specific genes and uncovered biological processes and functions relevant to *C*Lso acquisition and transmission into the host psyllid organs.

Whether the interaction between psyllid and the bacterium is of a beneficial nature or antagonistic can be debatable. In one scenario, the positive interactions between the psyllid and the bacterium could be crucial for their coexistence and confer an adaptive advantage over their natural predators. However, we and others have reported detrimental effects on the psyllid due to the presence of *C*Lso, such as a substantial reduction in reproductive fitness (Qiu and Scholthof, 2000; Montllor et al., 2002; Nachappa et al., 2014; Heyworth and Ferrari, 2016; Doremus and Oliver, 2017; Albuquerque Tomilhero Frias et al., 2020). The trade-off is strikingly not lethal, and the association has hence prevailed.

## Conclusion

In this study, we characterized the salivary glands- and ovaries-specific transcriptomes of *B. cockerelli* vectoring *C*Lso by employing the RNA-Seq approach and to identify molecular signatures of organ-specific gene expression and gene regulatory networks in the absence/presence of the bacterium. The study identified organ-specific gene expression patterns and unique biological processes affected in *B. cockerelli* organs in response to *C*Lso. For instance, biological processes related to neuronal transmission, cell adhesion, light stimulus, and respiration processes were primarily affected in the salivary glands. In contrast, cell division, DNA replication, translation, transcription regulation, and response to stress were affected in ovaries. These preferential biological processes may underpin the developmental and phenotypic responses of psyllids vectoring *C*Lso and contribute to the lateral and vertical transmission of the bacterium to plant hosts. Further experimental studies will be needed to determine the role of the candidate genes in these biological processes. Moreover, further understanding the complex antagonistic or beneficial interactions between psyllid and *C*Lso is crucial for developing robust pest/disease management strategies and reducing crop loss.

## Data Availability

The datasets presented in this study can be found in online repositories. The names of the repository/repositories and accession number(s) can be found in the article/**Supplementary Material**.
