# Supplementary material for: Insights into Bactericera cockerelli and Candidatus Liberibacter solanacearum interaction: a tissue-specific transcriptomic approach
Source: Front Plant Sci. 2024 Aug 30;15:1393994. doi: 10.3389/fpls.2024.1393994 (PMC11392735; doi:10.3389/fpls.2024.1393994)
Supplement: Supplementary file 1 [file DataSheet1.docx]

**Supplementary**

**Supplementary Table 1.** RNA-Seq data analysis of the psyllid ovaries and salivary glands under *C*Lso(-) and *C*Lso(+) conditions.

| **Samples** | **Total number of reads** | **Total number of high-quality reads** | **% high-quality reads** | **Total number of uniquely mapped reads** | **% uniquely mapped reads** |
| --- | --- | --- | --- | --- | --- |
| *C*Lso(-) ovaries rep 1 | 9526704 | 9053883 | 95.04 | 6106236 | 64.10 |
| *C*Lso(-) ovaries rep 2 | 11488811 | 10871034 | 94.62 | 7482107 | 65.13 |
| *C*Lso(-) ovaries rep 3 | 10090694 | 9586903 | 95.01 | 6608517 | 65.49 |
| *C*Lso(+) ovaries rep 1 | 9994908 | 9459339 | 94.64 | 6488186 | 64.91 |
| *C*Lso(+) ovaries rep 2 | 10928094 | 10342291 | 94.64 | 7198325 | 65.87 |
| *C*Lso(+) ovaries rep 3 | 9612705 | 9129612 | 94.97 | 6286482 | 65.40 |
| *C*Lso(-) salivary glands rep 1 | 9632525 | 9107750 | 94.55 | 5887599 | 61.12 |
| *C*Lso(-) salivary glands rep 2 | 11777179 | 10938046 | 92.87 | 6509825 | 55.27 |
| *C*Lso(-) salivary glands rep 3 | 9160592 | 8643706 | 94.36 | 4813311 | 52.54 |
| *C*Lso(+) salivary glands rep 1 | 10808005 | 10113470 | 93.57 | 6223007 | 57.58 |
| *C*Lso(+) salivary glands rep 2 | 9767527 | 9215273 | 94.35 | 5417249 | 55.46 |
| *C*Lso(+) salivary glands rep 3 | 8444782 | 7955594 | 94.21 | 4708527 | 55.76 |


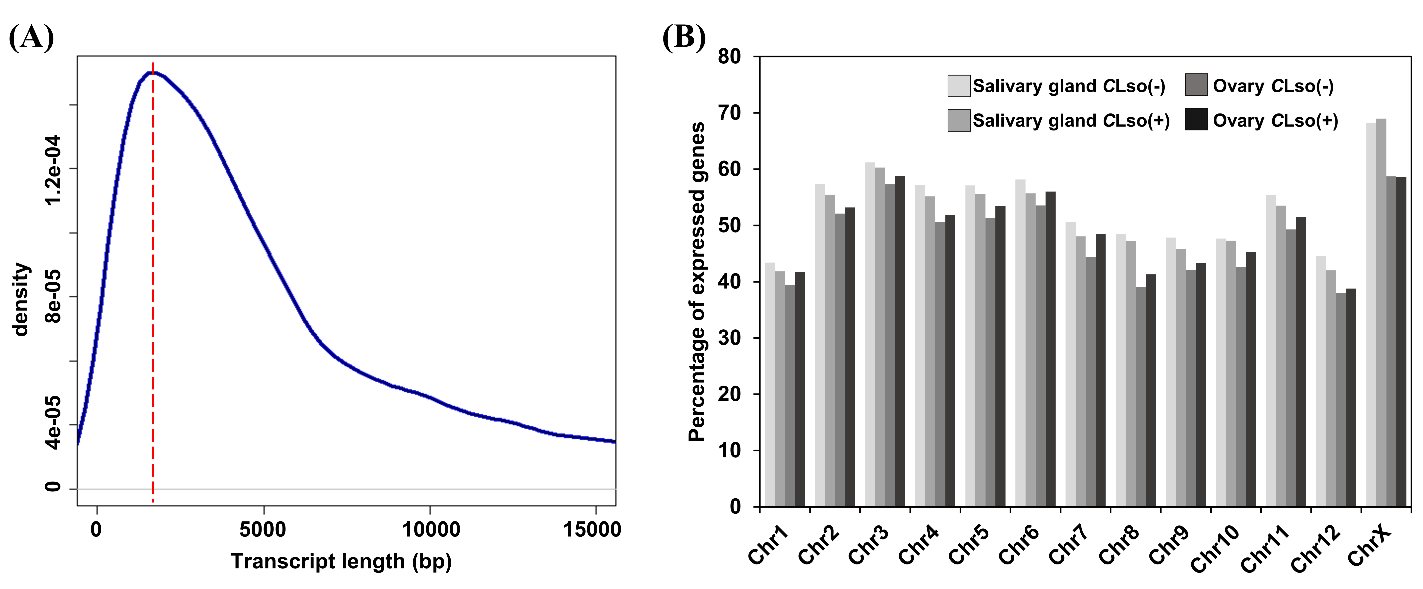


**Supplementary Figure 1. Length distribution and expression level of the psyllid genes across different chromosomes.** (A) The length distribution of genes is shown via kernel density plot. The peak of the density curve is marked with a red vertical line. (B) The percentage of expressed (≥0.5 FPKM) genes of the four samples representing the salivary glands and ovaries in control psyllids [*C*Lso(-)] and those carrying bacterium [*C*Lso(+)] across different chromosomes is shown via a bar plot.

**Supplementary Figure 2. Chromosome-wide expression level of the tomato-potato psyllid genes.** (A) The chromosome-wide frequency of all (outermost ring) and expressed genes (inner rings) in control psyllids [*C*Lso(-)] and those carrying bacterium [*C*Lso(+)] in the salivary glands and ovaries is shown via a Circos-plot. The scale at the bottom represents the frequency of all genes and expressed genes in a window size of 100 kb. (B) The expression level of genes across different chromosomes in the four samples given in (A) is shown via boxplot.

**Supplementary Figure 3. GO enrichment analysis of genes expressed in an organ-specific manner in the presence or absence of *C*Lso.** (A, B) Significantly enriched molecular function (A) and cellular component (B) GO terms of the psyllid genes expressed in an organ-specific manner (≥0.9 TSI) of the salivary glands and ovaries under *C*Lso(-) and *C*Lso(+) conditions are shown via bubble-plots. The scales represent the significance level (*P*-value) and number of genes in each enriched GO term.

**Supplementary Figure 4. GO enrichment analysis of specifically up- or downregulated genes in the presence of *C*Lso in the psyllid organs.** (A, B) Significantly enriched molecular function (A) and cellular component (B) GO terms for the sets of uniquely up- and downregulated genes in response to *C*Lso as compared to the uninfected control [*C*Lso(+)/*C*Lso(-)] in the ovaries and salivary glands are shown via bubble plots. The scales represent the significance level (*P*-value) and number of genes in each enriched GO term.

**Supplementary Figure 5. GO enrichment analysis of specifically up- or downregulated genes between the two psyllid organs in the absence or presence of *C*Lso.** (A, B) Significantly enriched molecular function (A) and cellular component (B) GO terms for the sets of uniquely up- and downregulated genes between the ovaries and salivary gland in the absence [*C*Lso(-)] or presence [*C*Lso(+)] of *C*Lso bacterium are shown via bubble plots. The scales represent the significance level (*P*-value) and number of genes in each enriched GO term.

**Supplementary Figure 6. Differentially expressed genes encoding transcription factors (TFs) between the ovaries and salivary glands in the tomato-potato psyllid.** (A) The number of genes encoding TFs exhibiting uniquely up- and downregulated in control psyllids [*C*Lso(-)] and those carrying bacterium [*C*Lso(+)] is shown via a Venn diagram. (B) The differential expression status of the TFs exhibiting unique up- and downregulation given in (A) is shown via heatmap. The scale at the bottom represents differential expression in log_2_ fold-change.


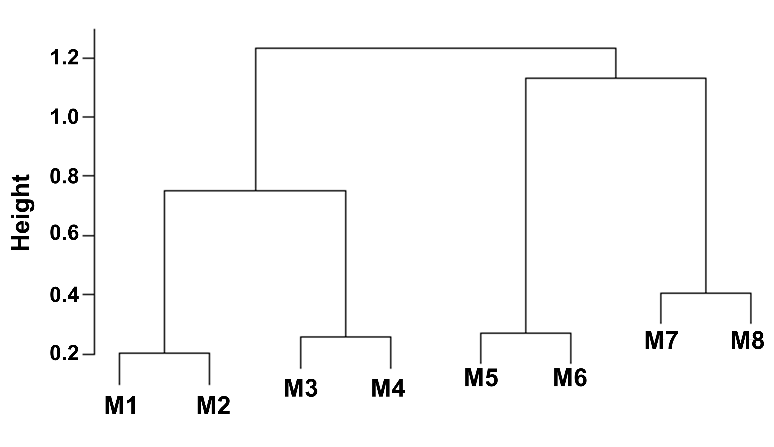


**Supplementary Figure 7. Phylogeny among the coexpressed modules.** Relationship among the eight modules detected via coexpression analysis of the salivary glands and ovaries in control psyllids [*C*Lso(-)] and those carrying bacterium [*C*Lso(+)] is shown via a dendrogram.

**Supplementary Figure 8. GO enrichment analysis of coexpressed genes distinguishing psyllid salivary glands and ovaries.** (A, B) Significantly enriched molecular function (A) and cellular component (B) GO terms of the coexpressed genes belonging to the M5 module and preferentially expressed in the psyllid salivary glands and ovaries in the presence or absence of the *C*Lso bacterium are shown via bubble plots. The scales represent the significance level (*P*-value) and number of genes in each enriched GO term.
